# *Porphyromonas gingivalis* bacteremia increases the permeability of the blood-brain barrier via the Mfsd2a/Caveolin-1 mediated transcytosis pathway

**DOI:** 10.1038/s41368-022-00215-y

**Published:** 2023-01-12

**Authors:** Shuang Lei, Jian Li, Jingjun Yu, Fulong Li, Yaping Pan, Xu Chen, Chunliang Ma, Weidong Zhao, Xiaolin Tang

**Affiliations:** 1 Department of Periodontics, School and Hospital of Stomatology, Liaoning Provincial Key Laboratory of Oral Disease, China Medical University, No. 117, Nanjing North Street, Heping District, Shenyang, China; 2 Department of Pediatric Dentistry, School and Hospital of Stomatology, Liaoning Provincial Key Laboratory of Oral Disease, China Medical University, No. 117, Nanjing North Street, Heping District, Shenyang, China; 3 Department of Preventive Dentistry, School and Hospital of Stomatology, Liaoning Provincial Key Laboratory of Oral Disease, China Medical University, Shenyang, China; 4 Department of Developmental Cell Biology, Key Laboratory of Cell Biology, China Medical University, 77 Puhe Road, Shenbei New District, Shenyang, China

**Keywords:** Bacterial infection, Health care

## Abstract

Bacteremia induced by periodontal infection is an important factor for periodontitis to threaten general health. *P. gingivalis* DNA/virulence factors have been found in the brain tissues from patients with Alzheimer’s disease (AD). The blood-brain barrier (BBB) is essential for keeping toxic substances from entering brain tissues. However, the effect of *P. gingivalis* bacteremia on BBB permeability and its underlying mechanism remains unclear. In the present study, rats were injected by tail vein with *P. gingivalis* three times a week for eight weeks to induce bacteremia. An in vitro BBB model infected with *P. gingivalis* was also established. We found that the infiltration of Evans blue dye and Albumin protein deposition in the rat brain tissues were increased in the rat brain tissues with *P. gingivalis* bacteremia and *P. gingivalis* could pass through the in vitro BBB model. Caveolae were detected after *P. gingivalis* infection in BMECs both in vivo and in vitro. Caveolin-1 (Cav-1) expression was enhanced after *P. gingivalis* infection. Downregulation of Cav-1 rescued *P. gingivalis*-enhanced BMECs permeability. We further found *P. gingivalis-*gingipain could be colocalized with Cav-1 and the strong hydrogen bonding between Cav-1 and arg-specific-gingipain (RgpA) were detected. Moreover, *P. gingivalis* significantly inhibited the major facilitator superfamily domain containing 2a (Mfsd2a) expression. Mfsd2a overexpression reversed *P. gingivalis-*increased BMECs permeability and Cav-1 expression. These results revealed that Mfsd2a/Cav-1 mediated transcytosis is a key pathway governing BBB BMECs permeability induced by *P. gingivalis*, which may contribute to *P. gingivalis*/virulence factors entrance and the subsequent neurological impairments.

## Introduction

Periodontitis is a chronic infectious disease that occurs in periodontal tissues, which eventually leads to tooth loss in adults. As the sixth largest disease worldwide, it is estimated that severe periodontitis affects 11% of the global population in 2021 and is associated with more than 50 types of systemic diseases.^1,2^ Recently, a systematic review reported periodontitis was associated with an increased risk of mortality due to cardiovascular diseases, cancer, coronary heart diseases, and cerebrovascular diseases.^3^ Among the closely related diseases, neurodegenerative diseases, especially Alzheimer’s disease (AD), have become the focus of research in the recent years.^4,5^

*Porphyromonas gingivalis* (*P. gingivalis*) is a keystone pathogen associated with periodontitis and has been proved to be a risk factor for AD.^6,7^ Recent studies have reported that *P. gingivalis* DNA or its virulence factors, including lipopolysaccharide and gingipain, have been detected in the brain tissues from patients with AD,^8,9^ and has been closely associated with AD pathological changes.^9^ However, how *P. gingivalis*/virulence factors enters the brain tissues remains unclear. The blood–brain barrier (BBB) is a key structural and functional barrier with low permeability, which is the first defense barrier to prevent *P. gingivalis* or its virulence factors from entering the brain tissues.^10^ The BBB is mainly composed of brain microvascular endothelial cells (BMECs), astrocytes, pericytes, and basement membrane. It exists between the blood circulatory system and central nervous system, which prevents peripheral toxic substances from entering the brain.^11^ Functional damage to the BBB is involved in cognitive dysfunction and neuronal loss.^12^ BBB breakdown is mainly characterized by increased bulk flow transcytosis (transcellular pathway) in BMECs and loss of intercellular junction (extracellular pathway) between adjacent BMECs.^13^ Recent studies have found that *P. gingivalis* virulence factor outer membrane vesicles (OMV), LPS, and gingipains could enhance the permeability of the in vitro BBB model by degrading the intercellular junction proteins.^14,15^ However, the effect of *P. gingivalis* infection on the transcytosis, or the transcellular pathway of BMECs permeability has not been elucidated.

The first step of transcytosis is endocytosis, a process of cellular uptake of extracellular materials within membrane-limited vacuoles. Our previous studies found that *P. gingivalis* could be internalized by epithelial cells and phagocytes and be embedded in vacuoles surrounded by single or double-layer membranes.^16,17^ Endocytosis is mainly mediated by clathrin-dependent and caveolae-dependent pathways. Caveolaes, the typical structure of transcytosis process, are flask-shaped organelles of approximately 50–100 nm that can regulate substance endocytosis, signal transduction, and cytoskeleton.^18,19^ Caveolin-1 (Cav-1), the most important structural protein of caveolae,^20^ has been shown to be critical in mediating the internalization of *P. gingivalis* in human oral epithelial cells.^21^ However, whether caveolae/Cav-1-mediated transcytosis is critical for *P. gingivalis* infection to promote the permeability of BMECs remains to be explored further.

Previous studies have established various *P. gingivalis* infection models in animals, such as the bacteremia model,^22^ the periodontitis model by the local ligation combined with besmearing bacteria^23^ or by the injection of virulence factors in the gingival papilla,^24^ to study the effects of *P. gingivalis* on the brain tissues. According to the concept of periodontal medicine, the primary pathogenic mechanism, under which periodontitis compromises systemic health, is that the subgingival plaque biofilm bacteria directly invade the blood vessels, causing bacteremia.^25^ Our previous research found that *P. gingivalis* bacteremia may reduce the learning and memory abilities of wild-type rats, and may reduce the protein expression levels of neuronal nuclei (NeuN) in the hippocampus, indicating that *P. gingivalis* bacteremia may result in neuron damage.^26^ Previous studies performed the high-intensity *P. gingivalis* injection via tail veins,^22,27,28^ however, the plaque accumulation of patients can significantly increase the prevalence of bacteremia following such daily oral activities as toothbrushing and the blood bacteria concentration is 0.97–32 CFU/mL,^29^ which is remarkably lower than the *P. gingivalis* intensity of bacteremia used in previous reports. Therefore, for the first time, we used the low-intensity *P. gingivalis* injection in rats in our previous study^26^ to simulate the possible bacteria intensity caused by oral daily activities, and we also found the damage of the neural cells.

To observe the effect of *P. gingivalis* on BBB permeability, we not only performed the in vivo experiments in rats with *P. gingivalis* bacteremia, but also established an in vitro BBB model in the present study. We further investigated the role and possible molecular mechanism of caveolae and Cav-1 in the regulation of BMECs permeability challenged with *P. gingivalis* infection. This study will provide potentially important evidence to determine the role of *P. gingivalis* bacteremia in increasing BBB permeability and subsequent neurological impairments.

## Results

### *Porphyromonas gingivalis* bacteremia enhanced the BBB permeability of rats

**Fig. 1:**
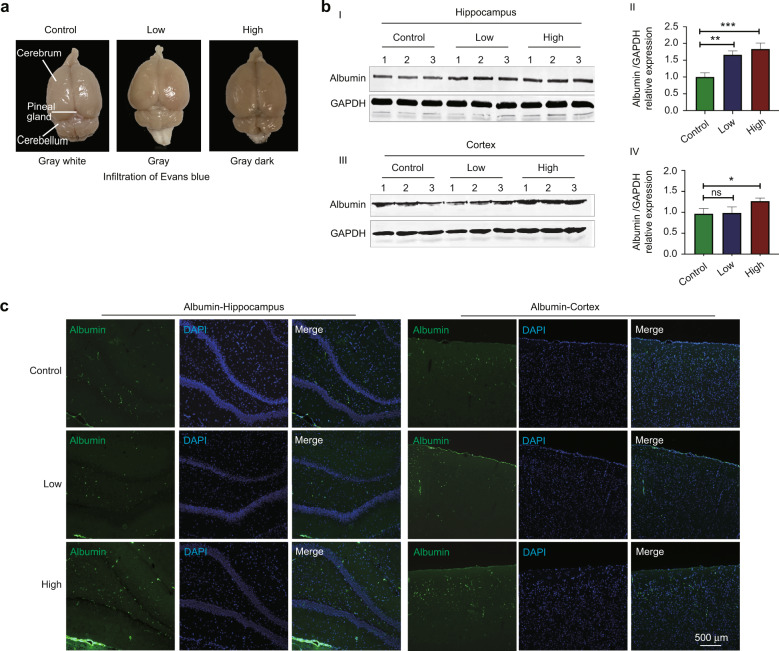
*Porphyromonas gingivalis (P. gingivalis)* bacteremia enhanced the blood–brain barrier (BBB) permeability in rats. **a** Evans blue staining was used to detect BBB permeability. **b** Western blot and quantification showed Albumin protein deposition in the hippocampus (I and II) and the cortex (III and IV) in the rat brain tissues with *P. gingivalis* bacteremia. ^*^P < 0.05, ^**^P < 0.01, ^***^P < 0.001 when compared to the control group. Values are expressed as the mean ± standard deviation. **c** Immunofluorescence showed Albumin (green) protein deposition increased both in the hippocampus and in the cortex of the rat brain with *P. gingivalis* bacteremia. Low: the low-intensity group. High: the high-intensity group. Scale bar, 500 μm. The results represent three independent experiments

To confirm whether *P. gingivalis* bacteremia can increase the BBB permeability in rats, Evans blue was used to detect the BBB integrity after *P. gingivalis* injection by vein tail for 8 weeks (refer to Supplementary Fig. 1). With *P. gingivalis* infection, more Evans blue infiltration were present, especially in the rat brains in the high *P. gingivalis* group compared to those in the control group. In detail, the color of the brains in the control group was gray white, and it turned into gray in the low *P. gingivalis* group while it was gray dark in the high *P. gingivalis* group. (Fig. 1a). Statistical analyses for the protein expression related to BBB breakdown demonstrated that *P. gingivalis* significantly increased expression of Albumin by 1.83-fold and 1.26-fold respectively in the hippocampus and cortex tissues (*P* < 0.001, *P* = 0.019) in the high intensity group. And in the low-intensity group, *P. gingivalis* only significantly increased the expression of Albumin by 1.62-fold in the hippocampus (*P* = 0.001) while had no effect in the cortex tissues (*P* = 0.838) (Fig. 1b). Immunofluorescence staining results also showed that Albumin protein levels significantly increased both in the hippocampus and cortex tissues in the high-intensity group (Fig. 1c).

### *Porphyromonas gingivalis* was able to pass through the BBB both in vivo and in vitro

**Fig. 2:**
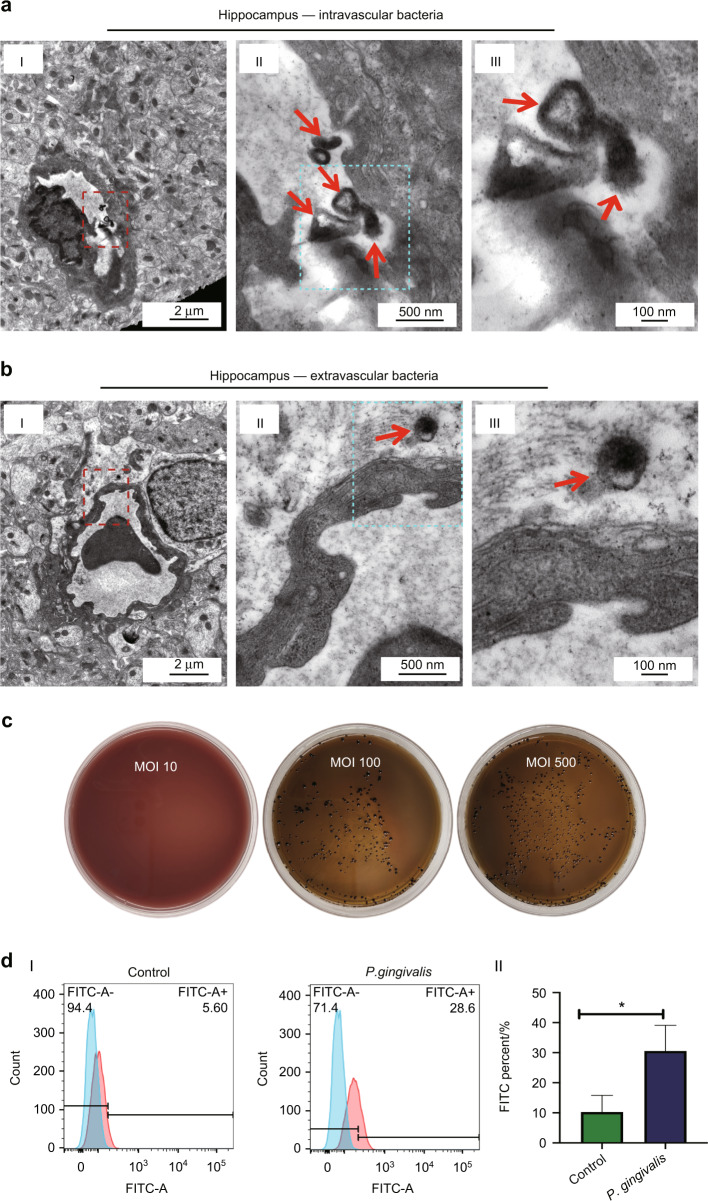
*Porphyromonas gingivalis (P. gingivalis)* passed through the blood–brain barrier (BBB) both in vivo and in vitro. **a** Intravascular *P. gingivalis-*like bacteria were detected in the rat hippocampus tissue with *P. gingivalis* bacteremia by transmission electron microscopy (TEM). Red arrows indicated *P. gingivalis-*like bacteria. I, II and III showed tissue photos with various magnification. **b** Extravascular *P. gingivalis-*like bacteria were detected in the rat hippocampus tissue with *P. gingivalis* bacteremia by transmission electron microscopy (TEM). Red arrows indicated *P. gingivalis-*like bacteria. I, II and III showed tissue photos with various magnification. Scale bars, 2 μm, 500 nm, and 100 nm. **c** The brain heart infusion agar anaerobic culture method showed that the bacteria in the lower chamber were detected in the multiplicity of infection (MOI) 100 and MOI 500 groups. **d** Flow cytometry (I) and quantification (II) showed fluorescein isothiocyanate (FITC)-positive cells increased in brain microvascular endothelial cells (BMECs) infected with *P. gingivalis* (MOI 100). ^*^P < 0.05 when compared to the control group. Values are expressed as the mean ± standard deviation. The results represent three independent experiments

To explore whether *P. gingivalis* was able to pass through the BBB, TEM was used to detect *P. gingivalis* bacteria in hippocampus. *P. gingivalis-*like bacteria were detected both intravascularly and extravascularly (Fig. 2a, b). To examine whether *P. gingivalis* enhanced the permeability of the BBB of the in vitro model, *P. gingivalis* (multiplicity of infection [MOI] 10, 100, or 500) was added into the upper chamber to treat BMECs for 24 h. *P. gingivalis* bacteria in the lower chamber were detected using the agar culture method. The results are shown in Fig. 2c. *P. gingivalis* colonies were detected at MOI 100 and 500, while no colonies were detected at MOI 10. Therefore, MOI 100 was used in the following experiments. FITC was used to detect the intercellular *P. gingivalis* as well as the permeability of BMECs by the flow cytometry method, which revealed that the percentage of FITC-positive BMECs significantly increased in the *P. gingivalis* (MOI 100) group (*P* = 0.026) compared with the control group (Fig. 2d).

### *Porphyromonas gingivalis* significantly increased the number of caveolar vesicles and caveolae-like structures while exerted no effect on the expression of Occludin in the hippocampus and cortex tissues of rats

**Fig. 3:**
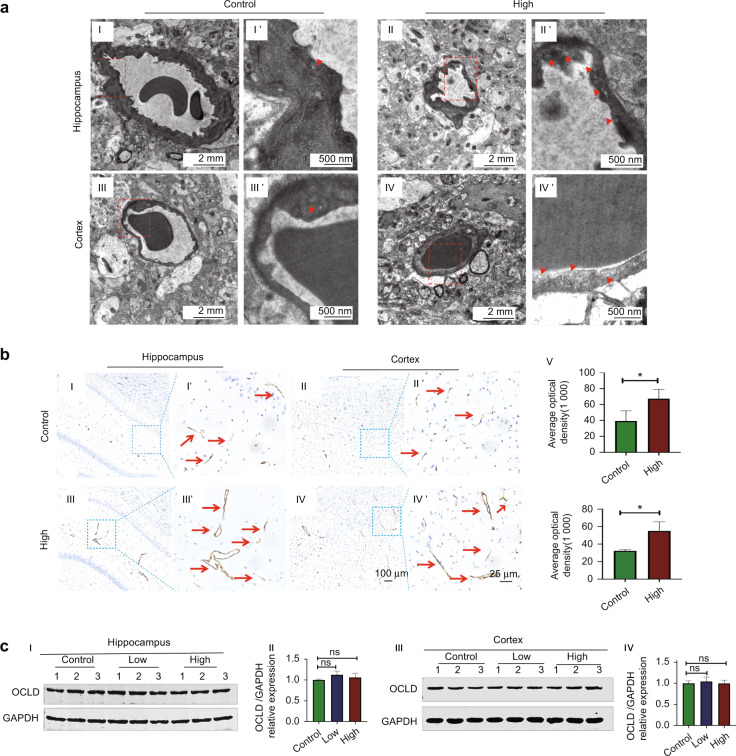
*Porphyromonas gingivalis (P. gingivalis)* bacteremia promoted the formation of caveolae-like structures but did not influence the intercellular junctions of microvessels in the rat brain tissues. **a** Representative images of ultrastructure in the hippocampus and cortex microvessels. Transmission electron microscopy (TEM) showed that the numbers of vesicles in the hippocampus and cortex microvessels increased in the high-intensity group compared to the control group, while no obvious damage was observed in the microvascular wall structure in the hippocampus and cortex tissues. (▲ indicates vesicle and caveolae-like structures. Scale bar, 2 μm and 500 nm). I and I': Hippocampus microvessels in the control group. II and II': Hippocampus microvessels in the high-intensity group. III and III': Cortex microvessels in the control group. IV and IV': Cortex microvessels in the high-intensity group. **b** Immunohistochemical staining and quantification showed that Caveolin-1 (Cav-1) expressions in the hippocampus (III and III') and cortex microvessels (IV and IV') in the high-intensity group significantly increased compared to those of the corresponding control groups (I, I', II, and II'). V and VI: Quantification of Cav-1 average optical density in the hippocampus and in the cortex. The red arrows indicated Cav-1 staining in BMECs of microvessels. **c** Western blot and quantification showed that the expression of Occludin (OCLD) protein did not significantly change in the brain tissues of rats with *P. gingivalis* bacteremia. I and II: The OCLD protein expression and quantification in the hippocampus. III and IV: The OCLD protein expression and quantification in the cortex. ^*^P < 0.05. High: the high-intensity group. Values are expressed as the mean ± standard deviation. The results represent three independent experiments

To further explore the specific mechanism of *P. gingivalis-*enhancing BBB permeability, mRNA transcriptomics high-throughput sequencing of bEnd.3 cells infected with *P. gingivalis* (MOI 100) were performed. As shown in Supplementary Figures, *P. gingivalis* significantly promoted the transcytosis pathway while had no effect on the extracelluar pathway. In animal experiments, we analyzed the subcellular structures of endothelial cells using TEM. We further found that the number of vesicles in the hippocampus and cortex tissues of the *P. gingivalis* infection increased significantly (Fig. 3a). All vesicles had a diameter range of 50–100 nm in line with that of normal caveolae (Fig. 3a). Cav-1 is a major marker and component of caveolar vesicles. Immunohistochemical staining showed that the protein level of Cav-1 in the hippocampus and cortex tissues significantly increased after *P. gingivalis* infection (*P* = 0.039, *P* = 0.02, Fig. 3b). Besides the transcytosis pathway, loss of intercellular junction is another important pathway for BBB breakdown. However, we further found that *P. gingivalis* had no effect on Occludin (OCLD) protein expression in the hippocampus and cortex tissues in rats of the high-intensity group (Fig. 3c). The above data indicated that *P. gingivalis* may enhance the BBB permeability mainly by the transcytosis pathway.

### Cav-1 regulated the internalization of *P. gingivali*s into BMECs

**Fig. 4:**
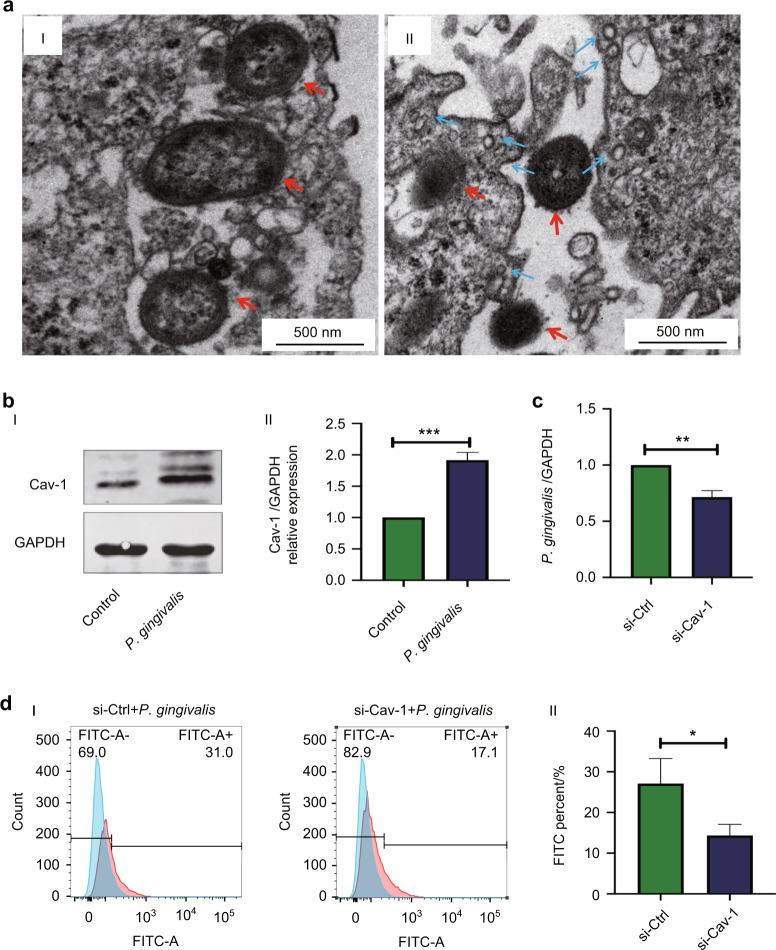
Caveolin-1 (Cav-1) regulated brain microvascular endothelial cells (BMECs) permeability after *Porphyromonas gingivalis (P. gingivalis)* infection. **a** Representative images of BMECs ultrastructure after *P. gingivalis* infection for 24 h. I: The status of *P. gingivalis* inside BMECs was observed by TEM. II: Caveolae-like structures of 50–100 nm were observed around the cell membrane of BMECs infected with *P. gingivalis*. Red arrows indicate intact *P. gingivalis*, and blue arrows indicate the caveolae-like structures. Scale bar, 500 nm. **b** Western blot and quantification (I and II) of the Cav-1 protein expression were evaluated in BMECs after *P. gingivalis* infection for 24 h. **c** RT-qPCR showed that the quantity of *P. gingivalis* ([MOI] 100) internalized into BMECs decreased after knockdown Cav-1. **d** Flow cytometry and quantification (I and II) showed that fluorescein isothiocyanate (FITC)-positive cells decreased after Cav-1 knockdown (si-Cav-1: knockdown with Cav-1). Values are expressed as the mean ± standard deviation. The results represent three independent experiments. ^*^P < 0.05, ^**^P < 0.01,^***^P < 0.001 when compared to the control group

We further explored the effect of caveolae/Cav-1 on the internalization of *P. gingivalis* into BMECs. TEM results showed that intact *P. gingivalis* was internalized into BMECs (Fig. 4a-I). We further found *P. gingivalis* infection enhanced caveolae formation on the surface of the cell membrane (Fig. 4 a-II). Simultaneously, *P. gingivalis* infection significantly increased Cav-1 protein expression by 1.9-fold in BMECs (*P* < 0.001) (Fig. 4b). The quantity of *P. gingivalis* in the BMECs^siCav-1^ group was significantly decreased by 0.71-fold than that in the si-control group (Fig. 4c). Flow cytometry revealed that the percentage of FITC-positive cells was lower in the BMECs^siCav-1^ group than in the si-control group (*P* = 0.03) (Fig. 4d).

### Cav-1 was able to bind with *Porphyromonas gingivalis-*RgpA

**Fig. 5:**
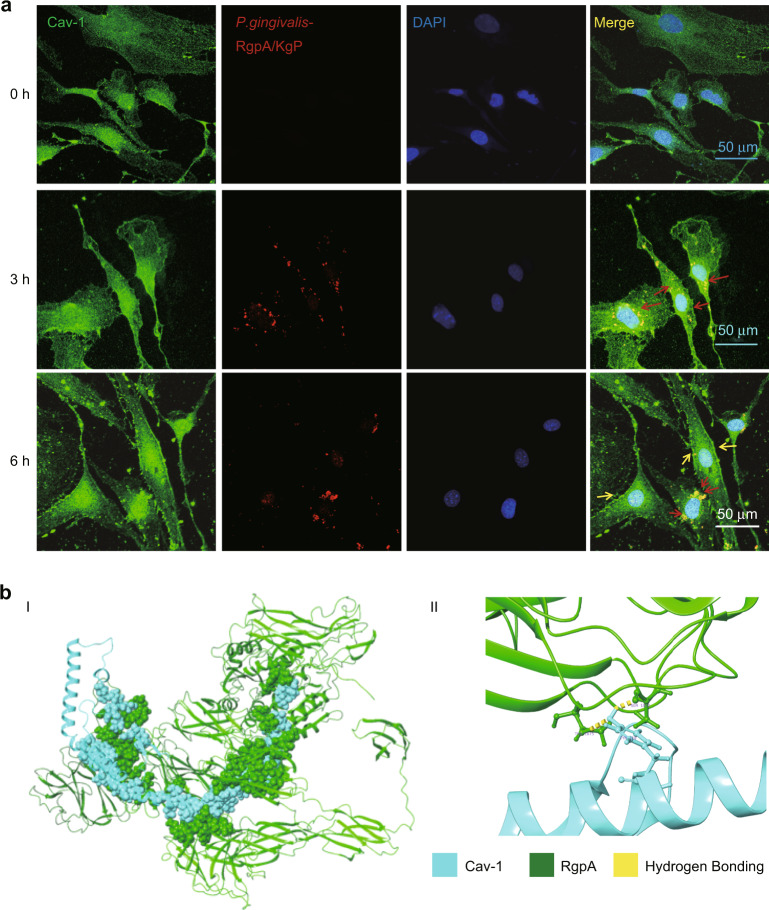
Cav-1 was able to bind with *Porphyromonas gingivalis (P. gingivalis)-gingipain*. **a** *P. gingivalis* promoted Cav-1 expression on surfaces of the cell membrane and *P. gingivalis/*gingipain (RgpA/Kgp) were colocalized with Cav-1. Representative images of Cav-1 in brain microvascular endothelial cells (BMECs) after infection with *P. gingivalis* of MOI 100 for 0, 3, and 6 h, respectively. (The red arrows indicate *P. gingivalis/*gingipain (RgpA/Kgp.) in BMECs. The yellow arrows indicate Cav-1 expression on the cell membrane surfaces. Scale bar, 50 μm. **b** I: Protein-protein docking of RgpA (green) and Cav-1 (blue). II: Ser1050, Thr1075 residuals of RgpA and Tyr118 residual of Cav-1 were combined by hydrogen bonding (yellow)

To further confirm the specific virulence factor of *P. gingivalis* interacting with Cav-1, CLSM was conducted and revealed that Cav-1 was uniformly expressed in the cells at the 0 h time point in BMECs infected with *P. gingivalis*. Three hours after infection, many bacteria were found in the cells, and the co-localization of *P. gingivalis/*gingipain and Cav-1 was also detected with further increasing Cav-1 expression on the cell membrane surfaces. Compared with the 3 h *P. gingivalis* infection, the number of bacteria in BMECs remained stable at 6 h, but Cav-1 expression continued to increase on the cell membrane (Fig. 5a). Immunoprecipitation (IP) and mass spectrometry were conducted and showed that *P. gingivalis-*Rgp was listed with the highest score among all the virulence factors of *P. gingivalis* interacting with Cav-1 (refer to Supplementary Fig. 2). We further assessed the protein–protein docking predictions of Cav-1 and RgpA. The interaction distance of RgpA at 1048–1075 residual and Cav-1 at 115–130 residual was less than 4 Å and the geometric complementarity was more than 50% (Fig. 5b), which indicated that the two proteins were relatively closely bound. In particular, Ser1050, Thr1075 residuals of RgpA and Tyr118 residual of Cav-1 were combined through hydrogen bonding (Fig. 5b).

### *Porphyromonas gingivalis* significantly inhibited the expression of Mfsd2a

**Fig. 6:**
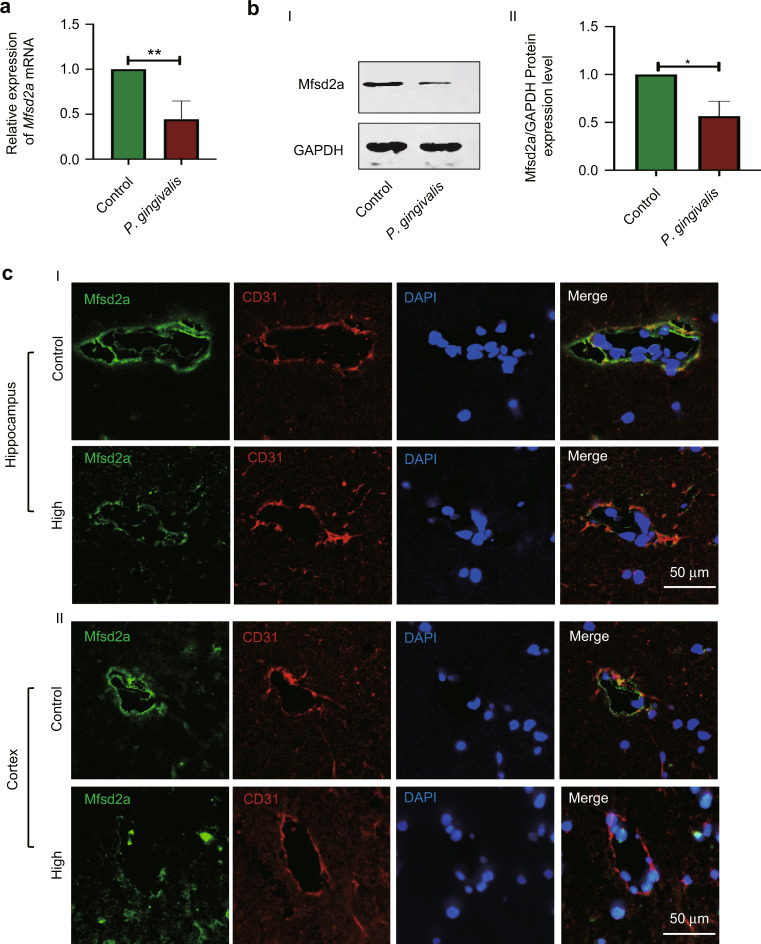
*Porphyromonas gingivalis (P. gingivalis)* significantly inhibited Mfsd2a expression. **a** RT-qPCR showed that *P. gingivalis* inhibited *Mfsd2a* gene expression in brain microvascular endothelial cells (BMECs). **b** Western blot (I) and quantification (II) showed Mfsd2a protein expression decreased in BMECs infected with *P. gingivalis* for 24 h. **c** Confocal laser microscopy revealed that Mfsd2a expression (green) in the microvasculature co-localized with CD31 (red) in the rat brain tissues with *P. gingivalis* bacteremia was sigificantly lower than that in the control group both in hippocampus and cortex tissues. I: Representative images of confocal laser microscopy in hippocampus. II: Representative images of confocal laser microscopy in cortex tissues. Scale bar, 50 μm. High: the high-intensity group. The results represent three independent experiments. ^*^P < 0.05, ^**^P < 0.01 when compared to the control group

To demonstrate the molecular mechanism of *P. gingivalis*-enhanced BBB permeability, high-throughput sequencing of BMECs infected with *P. gingivalis* (MOI 100) was performed. To analyze the specific molecular regulating permeability, cluster analysis showed that transcytosis-related gene *Mfsd2a* expression was downregulated significantly (refer to Supplementary Fig. 3). We further verified that *P. gingivalis* inhibited Mfsd2a mRNA expression by 0.44-fold (Fig. 6a) and protein expression by 0.57-fold (Fig. 6b) at 24 h in BMECs (*P* < 0.01, *P* = 0.04). Similarly, the confocal laser microscopy results also showed that *P. gingivalis* bacteremia significantly inhibited Mfsd2a expression in the microvessels of hippocampus and cortex tissues in the rats of the high-intensity group (Fig. 6c). The above data indicated that *P. gingivalis* was able to significantly inhibit Mfsd2a protein expression in BMECs both in vivo and in vitro.

### *Porphyromonas gingivalis* increased the permeability of brain microvascular endothelial cells by the Mfsd2a/Cav-1 axis

**Fig. 7:**
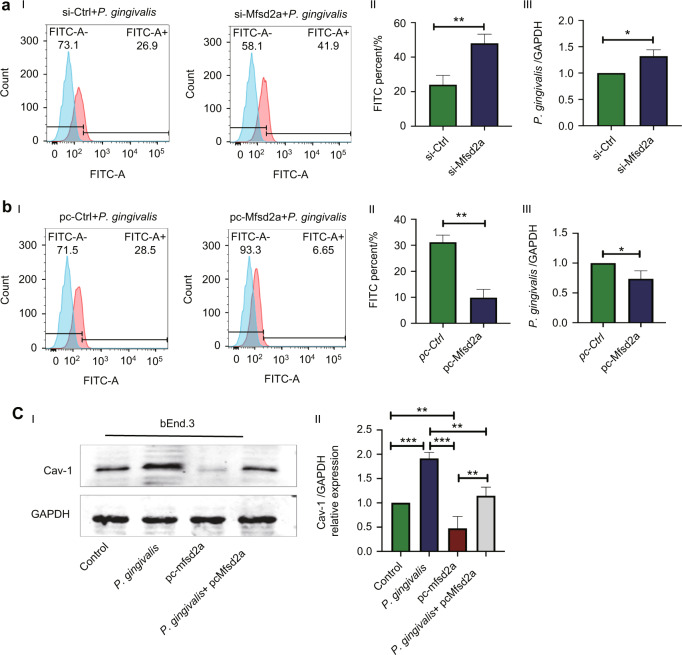
Mfsd2a negatively regulated the number of *Porphyromonas gingivalis (P. gingivalis)* internalized, cell permeability, and Cav-1 protein expression in brain microvascular endothelial cells (BMECs). **a** Mfsd2a knockdown significantly promoted *P. gingivalis* internalization into BMECs. I and II: Flow cytometry and quantification of fluorescein isothiocyanate (FITC)-positive cells. III: Quantity of *P. gingivalis* detected by RT-qPCR. **b** Mfsd2a overexpression significantly inhibited *P. gingivalis* internalization into BMECs. I and II: FITC-positive cells. III: Quantity of *P. gingivalis* detected by RT-qPCR. **c** Western blot (I) and quantification (II) showed that Cav-1 protein expression decreased after overexpressing Mfsd2a in BMECs infected with *P. gingivalis* for 24 h. Values are expressed as the mean ± standard deviation. The results represent three independent experiments. ^*^P < 0.05, ^**^P < 0.01,^***^P < 0.001 when compared to the control group. si, Mfsd2a knockdown with siRNA. pc, transfected with Mfsd2a plasmids

To confirm the role of Mfsd2a in BMECs permeability and Cav-1 expression, overexpressed plasmids and siRNA of Mfsd2a were transfected into BMECs (refer to Supplementary Fig. 4). After transfection with the Mfsd2a plasmid, the percentage of FITC-positive cells was remarkably lower in the BMECs^pcMfsd2a^ group than that in the pc-control group after 24 h *P. gingivalis* infection (*P* = 0.001). The quantity of *P. gingivalis* internalized into BMECs^pcMfsd2a^ was also significantly lower than that in the empty vector controls (*P* = 0.028, Fig. 7b). In contrast, the percentage of FITC-positive cells in the BMECs^siMfsd2a^ group was higher than that in the control group and the quantity of *P. gingivalis* in the BMECs^siMfsd2a^ group was higher than that in the si-control group (*P* = 0.005*, P* = 0.011, Fig. 7a). These results indicated that Mfsd2a was involved in *P. gingivalis-*enhanced cell permeability. Western blot showed that *P. gingivalis* enhanced Cav-1 protein expression in BMECs. Mfsd2a overexpression in BMECs decreased Cav-1 expression and attenuated *P. gingivalis*-increased Cav-1 expression (Fig. 7c).

## Discussion

Recent studies have demonstrated an association between periodontitis and cognitive impairment, such as AD.^4,8,30^ *P. gingivalis* and its virulence factors (LPS, gingipain) can be detected in the brain tissues of patients with AD,^8,31,32^ indicating that they can enter the brain tissue. BBB is the first defense barrier to prevent bacteria from entering. However, there are no relevant reports on the specific role of transcytosis in *P. gingivalis* breakthrough BBB. This study proposes, for the first time, that *P. gingivalis* infection enhances BBB permeability by regulating the Mfsd2a/Cav-1 transcytosis pathway.

In the present study, the high intensity of *P. gingivalis* at 10^8^ CFU per injection was chosen according to previous studies on AD.^22,27,28^ As for the low-intensity group, the bacteria intensity has been reported to be 0.97–32 CFU per mL,^29^ which can be converted into 10^3^ CFU per injection having been used in our previous study.^26^ Therefore, in the present study, 10^3^ CFU and 10^8^ CFU of *P. gingivalis* per injection were utilized.

This study confirms that *P. gingivalis* infection enhances BBB permeability. We found that in the high-intensity group, *P. gingivalis* bacteremia significantly enhanced BBB permeability so that the deposition of Evans blue dye and Albumin, a neurotoxic substance, significantly increased in the rat brain hippocampal and cortex tissues. In addition, in the low-intensity group, *P. gingivalis* bacteremia also significantly increased Albumin deposition in the hippocampal tissues, while had little effect on that in the cortex tissues and Evans blue dye deposition. The above data indicate that low-intensity *P. gingivalis* may increase the BBB permeability to some extent, which may further contribute to the subsequent neurological impairment in the long-term infection. However, since the bacteremia due to oral daily activities could be induced by mixed species of bacteria but not the single species of *P. gingivalis*, the actual pathological effects of bacteremia due to oral daily activities on BBB permeability should be carefully further explored and explained. Also, we detected *P. gingivalis*-like bacteria substances both within microvessels and in the brain parenchyma in the TEM sections from the rats with the high-intensity *P. gingivalis* injection. In addition, we found that *P. gingivalis* could pass through the in vitro BBB model and increase BMECs permeability. All the above data indicate that *P. gingivalis* may enhance BBB permeability and help the entrance of *P. gingivalis* /virulence factors into the brain tissues.

We further explored the possible mechanism by which *P. gingivalis* enhances BBB permeability in BMECs. Microorganisms can cause BBB disfunction and break through BBB to enter the central nervous system, which is an important etiological factor in AD. It has been reported that intestinal flora and respiratory flora can destroy and break through BBB through transcellular pathway or extracellular pathway.^33–35^ Our TEM results show that *P. gingivalis* bacteremia increased caveolae-like structures in the microvessels of the hippocampal and cortex tissues of rats. In addition, caveolae-like structures were observed in BMECs challenged with *P. gingivalis* infection. Studies have shown that Cav-1 can promote the transport of Albumin.^36^ Therefore, *P. gingivalis* bacteremia may promote the entry of Albumin into the brain through the BBB by promoting the Cav-1 expression and membrane localization in BMECs of the rats in the present study. Furthermore, Cav-1 knock-down significantly decreased the number of *P. gingivalis* internalized into BMECs. In addition, we detected the co-localization of *P. gingivalis* and Cav-1 with increased expression levels of Cav-1 on the cell membrane of BMECs. Similarly, phosphatidylserine Cav-1 has been found to be the key molecule for *P. gingivalis* to enter epithelial cells through the cavernous membrane, and Cav-1 and *P. gingivalis* exist at the same location on the cell surface.^37^ Although the classical theory believes that caveolae mainly mediate the internalization of nano-sized particles, studies have shown that caveolae can fuse into a structure larger than 100 nm and bacteria can enter the cell through caveolae. For example, Listeria can be mediated by multiple caveolae structures and then internalized into Madin–Darby canine kidney epithelial and HeLa cells. Based on the above evidence, we believe that *P. gingivalis* may promote caveolae formation and increase Cav-1 expression to enhance BBB permeability in BMEC of substances such as Albumin, and also the entry of *P. gingivalis* into the BMECs.

We further explored the virulent factors of *P. gingivalis,* which may mediate the interaction of *P. gingivalis* and Cav-1 and found that *P. gingivalis-*Rgp could be bound with Cav-1, which may partly contribute to *P. gingivalis* and Cav-1 interaction. Gingipain is one of the important virulent factor of *P. gingivalis*, which includes three kinds of cysteine proteinases, RgpA and arg-specific-gingipain B (RgpB) and lysine-specific-gingipain (Kgp).^38^ We used the Kgp/RgpA-specific antibody in CLSM observation and detected the co-localization of gingipain of *P. gingivalis* and Cav-1 in BMECs. Further IP assay showed that Rgp might be the key molecule bound with Cav-1. Therefore, we deduced that RgpA might mediate the binding between *P. gingivalis* and Cav-1. The subsequent protein docking assay provided the possible binding amino sequences of Cav-1 within its transmembrane region^39^ and those of RgpA within its hemagglutinin domain.^40^ Gingipain plays a central role in *P. gingivalis* colonization, inactivation of host defenses and the pathogenesis of AD.^9^ Therefore, we suggested that gingipain/ RgpA may be the critical molecule that mediates the interaction between *P. gingivalis* and Cav-1, which should be proved in the future experiments.

We further explored the possible molecular mechanism of *P. gingivalis*-induced caveolae formation. *P. gingivalis* bacteremia significantly inhibited Mfsd2a expression in rat brain tissue and BMECs. We further found that Mfsd2a inhibited Cav-1 expression and reduced the number of *P. gingivalis-*internalized cells. Mfsd2a is a key molecule for the formation and function of the BBB. In Mfsd2a knockout mice, transcytosis was significantly enhanced, but the TJ structure was not significantly changed.^41^ Mfsd2a can significantly inhibit the expression of caveolae-associated protein Cav-1 and cell membrane localization, thereby inhibiting cell cavernous formation.^42,43^ Based on the above evidence, we suggest that *P. gingivalis* may promote caveolae formation by inhibiting Mfsd2a expression and, in turn, increase BBB permeability in BMECs.

**Fig. 8:**
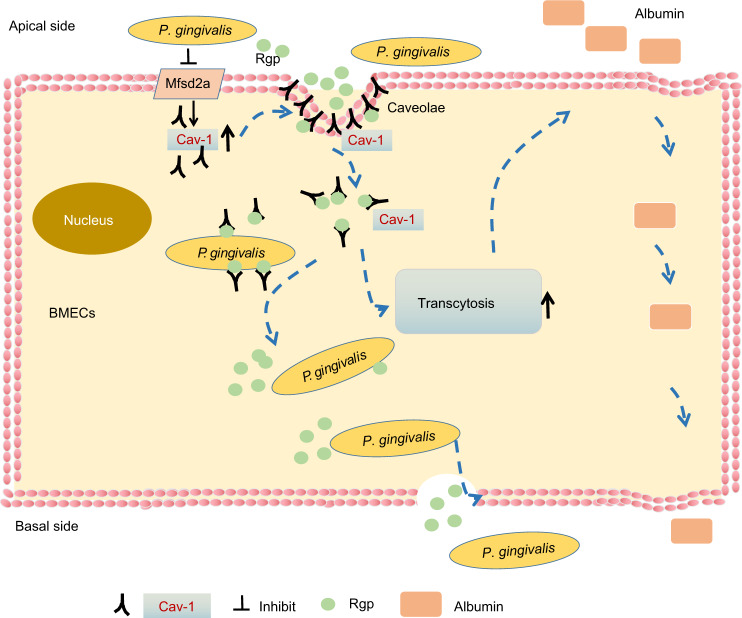
The schematic of the working hypothesis

In conclusion, our study indicates that *P. gingivalis* bacteremia may promote the permeability of BBB in BMECs by the Mfsd2a /Cav-1-mediated transcytosis pathway. In detail, decreased expression of Mfsd2a in BMECs by *P. gingivalis* infection may promote Cav-1 expression and caveolae formation, which in turn promotes transcytosis, thereby enhancing BBB permeability. The enhanced BBB permeability may promote the entrance of *P. gingivalis* and its virulence as well as neurotoxic substances such as Albumin into the brain tissues. Cav-1-mediated transcytosis plays a key role in the enhancement of BBB permeability by *P. gingivalis*. Furthermore, the interaction between Rgp and Cav-1 may take part in the endocytosis of *P. gingivalis* into BMECs. The hypothesis of the possible mechanism about the effect of *P. gingivalis* infection on BMECs permeability is concluded in Fig. 8. Therefore, it can be aimed at increasing Mfsd2a and inhibiting Rgp /Cav-1-mediated transcytosis to inhibit the injury of *P. gingivalis* on the BBB function. However, the virtual role of Rgp-Cav-1 interaction in *P. gingivalis*-induced increasing of BBB permeability in BMECs remains to be further studied in the future.

## Materials and methods

### Establishment of *P. gingivalis* bacteremia model

The *P. gingivalis* bacteremia model was established according to the methods described previously.^22,25–27^ This study was approved by the Laboratory Animal Welfare and Ethics Committee of China Medical University (Approval number: KT2021150). Eight-week-old healthy SD rats (body weight 180–220 g) were purchased from Changsheng Biotechnology Cable Company (Benxi City, Liaoning Province, China). The *P. gingivalis* ATCC 33277 strain was obtained from the American Tissue Culture Collection (Maryland, USA). The bacteria were maintained anaerobically at 37 °C on brain-heart-infusion (BHI) agar medium plates, supplemented with 5% sterilized and defibrinated sheep blood, 5 µL·mL^−1^ hemin, and 1 µL·mL^−1^ Vitamin K. The *P. gingivalis* was cultured in a liquid BHI medium for 16–18 h before experiments. All bacterial culture reagents were purchased from Aobo Bio-tech (Beijing, China). The experimental groups were injected intravenously with *P. gingivalis* through the tail vein three times a week for eight weeks. In the high-intensity group, the rats were injected with 200 µL PBS containing 10^8^ CFU *P. gingivalis*^22^ while in the low-intensity group, the rats were injected with 200 µL PBS containing 10^3^ CFU *P. gingivalis,*^26,29^ and in the control group the rats were injected with 200 µL PBS (*n* = 6 in each group).

### Transmission electron microscopy

The rats were injected with bacterial for 8 weeks by the tail vein. After deep anesthesia, the cerebral cortex and hippocampus brain tissues were taken out and fixed in 2.5% glutaraldehyde. The bEnd.3 were infected with *P. ginigivalis* for 24 h. The ultrathin sections were prepared, and the ultrastructure was observed with a transmission electron microscope (TEM, H7650, Hitachi, Tokyo, Japan).

### Western blot

Western blot was detected with BIO-RAD protein analysis system (BIO-RAD, USA). The primary antibodies were as follows: Cav-1 (Abcam, Cambridge, UK), Fibrinogen (Proteintech, Wuhan, China), Albumin (ABclonal, Wuhan, China), Occludin (Proteintech, Wuhan, China), anti-*P. gingivalis* (DSHB, Texas, USA), GAPDH (Proteintech, Wuhan, China). The fluorescent secondary antibody (Proteintech, Wuhan, China). Infrared fluorescence scanning imaging system (Odyssey CLx, LI-COR USA) was used to detect protein bands. ImageJ 1.52v software (NIH Image, Bethesda, MD, USA) was used for protein semi-quantitative analysis.

### Immunohistochemical

After deep anesthesia, the rats were perfused transcardially with pre-cold PBS and 4% paraformaldehyde solution (PFA) successively. Brains were removed and fixed in PFA at 4 °C. Paraffin sections were added with the primary antibody working solution of Cav-1 (Abcam, Cambridge, UK) overnight at 4 °C. Each section was added enzyme-labeled IgG polymer dropwise, incubated with DAB at room temperature.

### Immunofluorescence microscopy and laser confocal microscopy

After deep anesthesia, the rats were perfused transcardially with pre-cold PBS and 4% PFA successively. Brains were removed and fixed in PFA at 4 °C. Tissue paraffin sections were blocked and then incubated with the anti-rat Albumin antibody (ABclonal, Wuhan, China), anti-Mfsd2a antibody (Novus, Colorado, USA) and anti-CD31 antibody (Santa, Texas, USA). Cell samples were treated with *P. gingivalis* or with PBS, fixed with 4% PFA, treated with TritonX-100. The primary antibody was anti-*P. gingivalis* (DSHB, Texas, USA) and rabbit anti-mouse Cav-1 (Abcam, Cambridge, UK). The sections were incubated with the goat anti-mouse secondary antibody (Proteintech, Wuhan, China) and the goat anti-rabbit secondary antibody (Proteintech, Wuhan, China) for 2 h. The sections were stained with DAPI. Images were ultimately acquired with the aid of a fluorescence microscope (ECLIPSE, Nikon, Japan) and a laser-scanning confocal microscope (C2, Nikon, Japan).

### Establishment of the transwell BBB model in vitro and the permeability test

The mouse brain microvascular endothelial cell line bEnd.3 and the human astrocytoma cell line U87 were purchased from the Shanghai Cell Bank of the Chinese Academy of Sciences. Cells were cultured in DMEM medium supplemented with 10% fetal bovine serum (FBS) under the conditions of 37 °C with 5% CO_2_. The BBB is comprised of three kinds of specialized cells, including BMECs, astrocytes, and perivascular cells (pericytes). The co-culture transwell BBB model established by BMECs and astrocytes has been frequently reported in previous papers.^44,45^ bEnd.3 cells were incubated on the bottom of the upper transwell chamber (Corning 3402, Corning, USA), and U87 cells were incubated on the bottom of the lower transwell chamber. After 7 days, a certain amount of complete cell culture medium was added to the upper and lower chambers of the transwell unit to make the liquid level of the upper chamber higher than that of the lower chamber by 0.5 cm. After 4 h, if the original liquid level difference was maintained, the transwell BBB model should be considered to be successfully established.^46^ And then, *P. ginigivalis* ((MOI: 10, 100, 500) was added to the upper chamber for 24 h, and then the lower chamber liquid was aspirated for bacteria detection with the anaerobic culture method.

### Transfection assays

For transfection, the bEnd.3 cells were plated on six-well flatbottom plates at a seeding density of 2 × 10^5^ and grew to 80% confluence. The Mfsd2a plasmids were transfected into bEnd.3 cells for 24 h. The Mfsd2a siRNA was transfected into bEnd.3 cells for 24 h. The cells treated with empty vectors or scrambled siRNA were used as the negative control.

### Flow cytometry

For flow cytometry, cells were divided into six groups and compared with each other in different experiments: control group, *P. gingivalis*, empty vector plasmid transfection+ *P. gingivalis*, Mfsd2a plasmid transfection + *P. gingivalis*, scrambled siRNA transfection + *P. gingivalis*, and Mfsd2a-siRNA transfection + *P. gingivalis*. After transfection, *P. gingivalis* was added to infect the BMECs for 24 h. Fluorescein isothiocyanate (FITC) dilution was then added to the wells for 10 min. The fluorescence intensity of FITC of the cells was detected by flow cytometry (FACS, Becton-Dickinson, Islandia, NY, USA) and analyzed using FlowJovX0.7 software.

### Quantitative real-time polymerase chain reaction

In this study, we established a bacterial internalization model by the antibiotics protection assay. The bEnd.3 cells were infected with *P. gingivalis* with a specific multiplicity of infection (MOI) for 6 h. Then cells were washed three times with PBS and were further incubated in the culture medium containing 300 µg/mL of gentamicin and 200 µg·mL^−1^ of metronidazole (Sigma, St. Louis, MO, USA) for 2 h. Total RNA was extracted from cells using TRIzol reagent (Invitrogen Life Technologies, Gaithersburg, MD, USA) according to the manufacturer’s instructions. Real-time PCR analyses were conducted on an ABI Prism 7500 Sequence Detection System (Applied Biosystems, Foster City, CA, USA) in combination with a SYBR Premix Ex TaqTM II PCR Master Mix Reagents kit (Takara Bio, Inc., Dalian, China). *P. ginigivalis* 16s RNA primer sequence: Forward Primer: AGGCAGCTTGCCATACTGCG, Reverse Primer: ACTGTTAGCAACTACCGATG. Fold changes were calculated through relative quantification (2^−△△CT^) as previously reported. The quantity of *P. gingivalis* was displayed as the relative ratio to GAPDH expression level according to the methods reported in our previous studies.^16,47^ Each experiment was performed in triplicates.

### Immunoprecipitation and mass spectrometry analysis

The immunoprecipitation was conducted as the kit instructions (Takara Bio, USA). Briefly, protein lysates were centrifuged, and the supernatant was removed and kept. Incubate the recommended amount of Cav-1 antibody overnight. The eluted antibody-protein complex with the neutralization buffer in the tube was collected. Subsequently, the sample was analyzed by mass spectrometry (Beijing Protein Innovation Co. Ltd, Beijing, China).

### Protein–protein docking

The protein–protein docking was conducted by APExBIO Technology LLC (Shanghai, China). Cav-1 and *P. gingivalis-*arg-specific-gingipainA (RgpA) protein structures were queried from the UniProt database and the structure files were saved in pdb format. The pdb files were imported into the Maestro docking software. Cav-1 and RgpA were set as a ligand and the receptor, respectively, for the docking parameters. Thirty docking poses were output after docking and the Protein Interaction docking complex was used for interaction analysis. Protein docking data were analyzed and the graphs were drawn.

### Statistical analysis

Normally distributed data were expressed as the means ± standard deviation (SD). Differences among the three group were analyzed by multiple comparisons using one-way analysis of variance (ANOVA). Differences among the two groups were analyzed by an independent two-tailed *t* test. SPSS 22.0 software package (SPSS Inc., Chicago, IL, USA) was used to perform the analysis. *P* < 0.05 was considered to be statistically significant.

## Supplementary information

supplemental material
