# Supplementary material for: Porphyromonas gingivalis bacteremia increases the permeability of the blood-brain barrier via the Mfsd2a/Caveolin-1 mediated transcytosis pathway
Source: Int J Oral Sci. 2023 Jan 12;15:3. doi: 10.1038/s41368-022-00215-y (PMC9834243; doi:10.1038/s41368-022-00215-y)

Supplementary results

Figure1.

Schematic of the animal experiment design.

Figure2.

(a) Total ion chromatograms of mass spectrometry analysis of the proteins. (b) Secondary mass spectrum of P. gingivalis-Rgp . (c) The binding sites of RgpA and Cav-1.

Figure3.

To demonstrate the molecular mechanism of *P. gingivalis*-enhanced BBB permeability, high-throughput sequencing of BMECs infected with *P. gingivalis* (MOI 100) was performed. According to the differential gene screening ($|Log_2FC| \geqslant 1$, $P < 0.05$, FDR < 0.05), there were 1661 differential genes, including 983 upregulated and 678 downregulated genes after *P. gingivalis* treatment compared with control group (Fig. 2a). Gene Ontology (GO) assignments were used to assign functional classifications to the differentially expressed genes (DEGs) . All DEGs were annotated with 20 functional terms and categorized as biological process, cellular component, or molecular function (Fig. 2b). Cluster of orthologous group function annotation analysis showed that the DEGs were mainly enriched in intracellular transport, secretion, and vesicle transport pathways (Fig. 2c). To analyze the specific molecular regulating permeability, cluster analysis showed that transcytosis-related gene *Mfsd2a* expression was downregulated significantly (Fig. 1D).

Figure4.

To confirm the role of Mfsd2a in BMECs permeability and Cav-1 expression, overexpressed plasmids and siRNA of Mfsd2a were transfected into BMECs. Compared with the control group, Mfsd2a protein expressions in the BMECs^{pcMfsd2a} (Fig.2a) and BMECs^{SiMfsd2a} groups (Fig.2b) were significantly higher and lower at 24 h, respectively.

Figure1

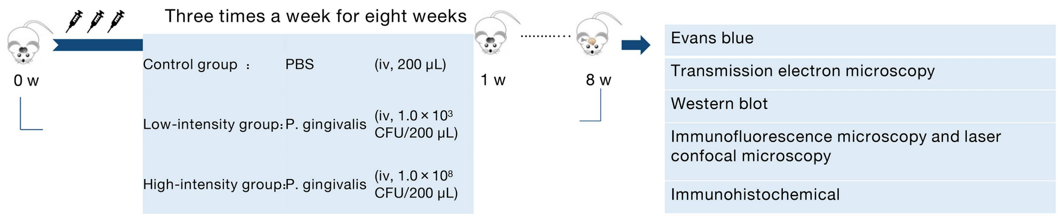

Figure2

a **b**

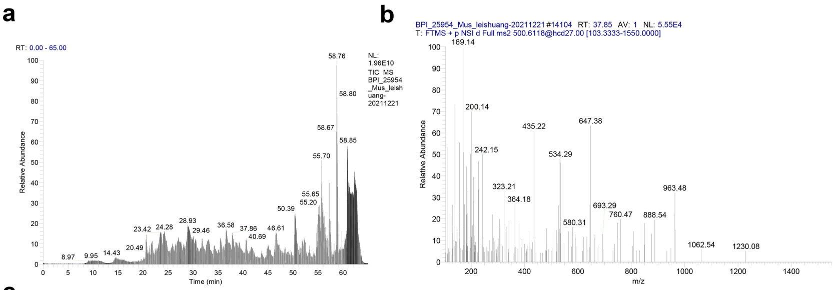

c

Protein	binding site	The sequence
RgpA	1048-1075	YASEHYAVYASSTGNDASNFTNALLEET
Cav-1	115-130	WGIYFAILSFLHIWAV

Figure3

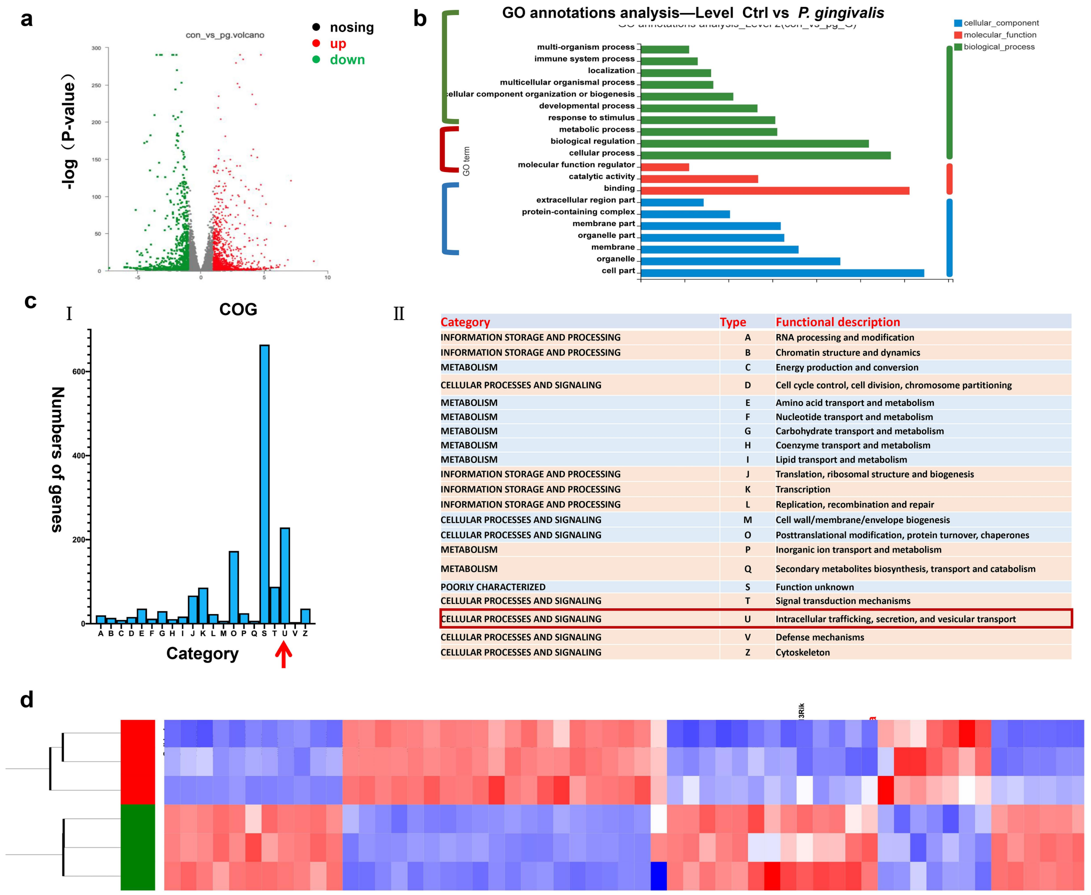

Category	Type	Functional description
INFORMATION STORAGE AND PROCESSING	A	RNA processing and modification
INFORMATION STORAGE AND PROCESSING	B	Chromatin structure and dynamics
METABOLISM	C	Energy production and conversion
CELLULAR PROCESSES AND SIGNALING	D	Cell cycle control, cell division, chromosome partitioning
METABOLISM	E	Amino acid transport and metabolism
METABOLISM	F	Nucleotide transport and metabolism
METABOLISM	G	Carbohydrate transport and metabolism
METABOLISM	H	Coenzyme transport and metabolism
METABOLISM	I	Lipid transport and metabolism
INFORMATION STORAGE AND PROCESSING	J	Translation, ribosomal structure and biogenesis
INFORMATION STORAGE AND PROCESSING	K	Transcription
INFORMATION STORAGE AND PROCESSING	L	Replication, recombination and repair
CELLULAR PROCESSES AND SIGNALING	M	Cell wall/membrane/envelope biogenesis
CELLULAR PROCESSES AND SIGNALING	O	Posttranslational modification, protein turnover, chaperones
METABOLISM	P	Inorganic ion transport and metabolism
METABOLISM	Q	Secondary metabolites biosynthesis, transport and catabolism
POORLY CHARACTERIZED	S	Function unknown
CELLULAR PROCESSES AND SIGNALING	T	Signal transduction mechanisms
CELLULAR PROCESSES AND SIGNALING	U	Intracellular trafficking, secretion, and vesicular transport
CELLULAR PROCESSES AND SIGNALING	V	Defense mechanisms
CELLULAR PROCESSES AND SIGNALING	Z	Cytoskeleton

Figure4

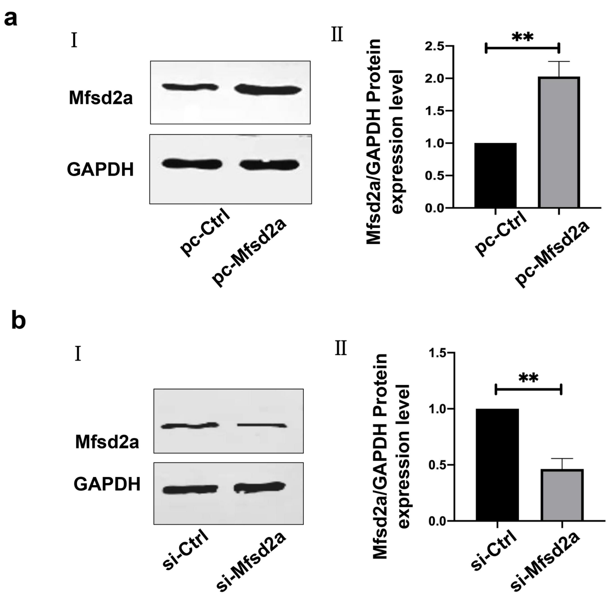

Supplement: Supplementary file 1 — supplemental material [file 41368_2022_215_MOESM1_ESM.pdf]
